# Probiotics and gastrointestinal disease: successes, problems and future prospects

**DOI:** 10.1186/1757-4749-1-19

**Published:** 2009-11-23

**Authors:** Eamonn P Culligan, Colin Hill, Roy D Sleator

**Affiliations:** 1 Department of Biological Sciences, Cork Institute of Technology, Rossa Avenue, Bishopstown, Cork, Ireland; 2 Alimentary Pharmabiotic Centre, University College Cork, Western Road, Cork, Ireland; 3 Department of Microbiology, University College Cork, Western Road, Cork, Ireland

## Abstract

Gastrointestinal disease is a major cause of morbidity and mortality worldwide each year. Treatment of chronic inflammatory gastrointestinal conditions such as ulcerative colitis and Crohn's disease is difficult due to the ambiguity surrounding their precise aetiology. Infectious gastrointestinal diseases, such as various types of diarrheal disease are also becoming increasingly difficult to treat due to the increasing dissemination of antibiotic resistance among microorganisms and the emergence of the so-called 'superbugs'. Taking into consideration these problems, the need for novel therapeutics is essential. Although described for over a century probiotics have only been extensively researched in recent years. Their use in the treatment and prevention of disease, particularly gastrointestinal disease, has yielded many successful results, some of which we outline in this review. Although promising, many probiotics are hindered by inherent physiological and technological weaknesses and often the most clinically promising strains are unusable. Consequently we discuss various strategies whereby probiotics may be engineered to create designer probiotics. Such innovative approaches include; a receptor mimicry strategy to create probiotics that target specific pathogens and toxins, a patho-biotechnology approach using pathogen-derived genes to create more robust probiotic stains with increased host and processing-associated stress tolerance profiles and meta-biotechnology, whereby, functional metagenomics may be used to identify novel genes from diverse and vastly unexplored environments, such as the human gut, for use in biotechnology and medicine.

## Introduction

The first suggestion of the health benefits of probiotics dates back to the early 20^th ^century, when Russian scientist Eli Metchnikoff attributed the long life of Bulgarian peasants to their consumption of fermented milk containing lactic acid bacteria (LAB) and believed that *'the dependence of the intestinal microbes on the food makes it possible to adopt measures to modify the flora in our bodies and to replace the harmful microbes with useful microbes' *[1]. While early research yielded poor results and received little attention, in the last 20 years probiotic research has again come to the forefront of scientific research with significant progress being made in the development of clinically effective probiotic strains. Probiotics are defined as 'live microorganisms which when administered in adequate amounts confer a health benefit on the host' [2]. There is an increasing body of evidence to suggest that probiotics can be used in the treatment and prevention of infections and chronic inflammatory disorders of the gastrointestinal tract.

### Use of probiotics in the treatment and prevention of gastrointestinal disease

#### Diarrheal disease

Each year gastrointestinal infections are responsible for significant morbidity and mortality worldwide. The World Health Organisation (WHO) estimates there to be more than four billion episodes of diarrheal disease annually, while there were 2.2 million deaths attributable to diarrheal disease in 2004, making it the fifth leading cause of death at all ages worldwide [3]. Probiotics have been used in the treatment and prevention of many forms of diarrheal disease.

(i) Rotavirus diarrhea: A double-blind randomized placebo-controlled trial of 230 children using the probiotic formulation VSL#3 was found to significantly reduce stool frequency and requirement for oral rehydration salts (ORS) compared to the placebo group, resulting in reduced recovery time [4]. In another study, administration of *Lactobacillus rhamnosus GG *to infants admitted to hospital with non-diarrheal complaints, reduced the risk of both nosocomial diarrhea and symptomatic rotavirus gastroenteritis [5].

(ii) Travellers' diarrhea: Travellers diarrhea is a frequent problem among travellers to foreign countries, especially in South America, South East Asia and Africa. A meta-analysis of 940 studies by (12 of which met the inclusion criteria; various database searches from 1977 to 2005 to include randomization, controlled, blinded efficacy trials in humans from peer reviewed journals) concluded that probiotics are safe and effective for prevention of traveller's diarrhea [6]. However, contradictory results are often seen in studies of this kind due to differences in populations studied, type of probiotics used and the duration of treatment. Also, a number of factors may affect the efficacy of treatment such as travel destination, probiotic viability during the trip and traveller compliance with treatment.

(iii) Antibiotic-associated diarrhea (AAD): AAD is often seen in patients receiving antibiotic therapy which results in the suppression of the normal host gastrointestinal microflora, thus facilitating the overgrowth of enteropathogens, which can cause diarrhea and colonic inflammation (colitis). In extreme cases, *Clostridium difficile *can often cause pseudomembranous enterocolitis [7], which can be fatal. A clinical trial by Ruszczyński and co-workers assessed the efficacy of three *L. rhamnosus *strains in the prevention of AAD [8]. In this double-blind, randomized, placebo-controlled trial of 240 children, 20 patients in the placebo group had diarrhea compared to nine in the probiotic group. Furthermore, AAD diarrhea was seen in nine placebo patients compared to three of those administered the probiotic preparation. Also, various meta-analyses have shown probiotics to be successful in the prevention of AAD [9,10].

(iv) *Clostridium difficile *is a major cause of nosocomial infection with symptoms ranging from mild diarrhea to severe pseudmembranous enterocolitis, sepsis and death [11]. Overall, adequate evidence is lacking to recommend the use of probiotics in the prevention or treatment of *C. difficile*. There have been some promising studies using the probiotic yeast, *Saccharomyces boulardii *[12,13] however more research is needed encompassing large, standardised clinical trials with different probiotic strains.

### Necrotizing enterocolitits (NEC)

NEC is a serious gastrointestinal condition typically seen in premature infants. Symptoms include abdominal distension, bloody stool, and lethargy. A number of studies have demonstrated that probiotic therapy reduced both the incidence and severity of NEC in a study of very low birth weight infants [14,15].

### Inflammatory bowel disease (IBD)

IBD encompasses chronic inflammatory conditions of the gastrointestinal tract, characterized by unpredictable and spontaneous periods of remission and relapse. The most common types of IBD are ulcerative colitis (UC) and Crohn's disease (CD), with the prevalence of IBD estimated to be 1.4 million in the United States and 2.2 million in Europe [16]. While the precise aetiology of IBD is unknown, genetic susceptibility, imbalances or disruption to the commensal host microflora [17] especially a reduction in the Firmicutes phylum [18] and an abnormal intestinal immune response [19,20] are thought to play an important role in disease manifestation. Consequently, probiotics have been utilised in an attempt to re-establish the balance of the host microflora and attenuate an aberrant immune response. In a trial of UC patients investigating the effect of an oral capsule containing *Bifidobacteria *following treatment with sulfasalazine and glucocorticoids (a standard therapy for UC) it was found that 93.3% of patients in the placebo group suffered a relapse compared to only 20% in the probiotic group. A significant reduction in inflammation was also seen in those administered the probiotic compared to the control group [21]. A recent study has shown promising potential for the use of *Faecalibacterium prausnitzii *as a probiotic with anti-inflammatory properties in the treatment of CD [22]. This bacterium was found in lower numbers in patients with recurrent CD. *F. prausnitzii *and its supernatant was found to have anti-inflammatory effects both *in vitro *and *in vivo*, inducing interleukin 10 (IL-10) production in peripheral blood mononuclear cells (PBMC's), reducing IL-8 and NF-κβ (pro-inflammatory compounds) in Caco-2 cell lines and attenuating the severity of induced colitis in mice [22]. There is, as yet, little evidence documenting the effectiveness of probiotics in the treatment of CD and as such further research is required. Shanahan [23] suggests that differences in the composition of the host microflora and the locations of CD-associated lesions respectively along the GI tract may indicate one probiotic strain is not sufficient to exert a beneficial effect in different patients. Furthermore, the author poses the question of whether researchers are using the correct probiotic, at a high enough dose and for the correct indication. Overall, only a limited number of studies are available and often results are conflicting, but there is sufficient evidence to warrant further research. Future studies need to be randomized, double-blind placebo-controlled trials encompassing large subject bases and possibly using combination therapies with more than one probiotic strain. Probiotics have also been used in the treatment and prevention of other gastrointestinal disorders including; irritable bowel syndrome (IBS) [24] and *Helicobacter pylori *associated infection (for a review see; [25]), as well as in non-gastrointestinal conditions such as urinary tract infections (UTI's) [26] and atopic diseases [27].

### Selection criteria and characteristics of probiotics

Before bacteria can be considered for use as probiotics, it is recommended that they meet certain selection criteria and possess a number of intrinsic physico-chemical characteristics outlined in a joint report by the FAO and WHO in 2002 [28]. The report sets out a number of guidelines that should be followed so as to standardise procedures when assessing probiotics for use in foods and validating health claims thereof. The guidelines include: probiotic health effects are usually strain specific [29] so it is important to characterize probiotics to the strain level and with current nomenclature and subsequently deposit them in an international culture collection. It was recommended that *in vitro *tests be carried out before any subsequent *in vivo *animal or human trials were initiated. Furthermore, these tests require substantiation with *in vivo *performance. Common *in vitro *tests performed on probiotics include resistance to gastric acidity and bile, adherence to mucus and/or human epithelial cells, bile salt hydrolase activity, reduction of pathogen adherence to surfaces and antimicrobial activity against pathogenic bacteria such as *Heliocbacter pylori*, *Salmonella *sp., *Listeria monocytogenes *and *Clostridium difficile*. The report also outlines some safety considerations when dealing with probiotics. While probiotic bacteria, as a group, are generally regarded as safe (GRAS) organisms, safety tests should include the determination of antibiotic resistance profiles, evaluation of certain metabolic activities such as D-lactate production and bile salt deconjugation (an undesirable action in the small bowel), assessment of side effects in humans trials, post market epidemiological surveillance of adverse effects in consumers, toxin production and haemolytic activity. Furthermore, animal trials should be undertaken, where possible and appropriate, before commencing human studies. There have been very few reports of possible systemic infections attributed to probiotics [30-32] and in those rare instances, all occurred in patients with serious underlying medical conditions. However, demonstration of lack of infectivity in immuno-compromised animals is also desirable to reinforce the safety profile of the probiotic strain. Large human clinical trials with probiotics are sorely lacking and the report recommends double-blind, randomized, placebo-controlled trials for humans with a sufficiently large participant base and preferably that the trial be repeated by an independent laboratory to confirm the outcome. The full report can be downloaded at: ftp://ftp.fao.org/es/esn/food/wgreport2.pdf.

### *In vivo *survival

Following oral administration, probiotics must survive transit through the gastrointestinal tract, facing host-associated stresses such as the low pH environment of the stomach (which can be as low as pH 1.5 when fasting) [33] as well as bile and elevated osmolarity in the intestine. Once in the gut they must be able to colonise and proliferate and exert a beneficial effect on the host. In addition to host-associated stresses, probiotics encounter technological stresses during processing, formulation and packaging and must survive in sufficient numbers for an extended period of time during their shelf-life, often at refrigeration temperatures. They should also possess good organoleptic properties and be phage resistant [34]. Potential probiotic strains need to be both physiologically and technologically robust, but even the toughest strains are restricted in the variety of food applications to which they can be applied. In addition the most promising and clinically relevant probiotics are unfortunately often rendered unusable due to their physiological and technological fragility [34]. Pre-exposing probiotic strains to stresses such as sodium chloride (NaCl), elevated temperature, bile and low pH can increase their survival and viability [35,36]. This pre-exposure to sub-lethal stresses can significantly increase their survival following subsequent exposure to lethal stress. It has been demonstrated that exposing *Bifidobacterium adolescentis *to 47°C for 15 minutes prior to a lethal heat shock increased the strain's heat tolerance 128-fold [37]. Furthermore it has been shown that acid-resistant strains can be produced by subjecting acid-sensitive strains to prolonged exposure (16 hours) to pH2.0 [38]. The acid-resistant derivatives were seen to obtain a cross-protective effect from acid exposure, growing better in the presence of both bile and NaCl, whilst also having an increased fermentative ability and enzymatic activity. Such treatments however can result in a significant decrease in cell yield, as well as cellular activity and process volumetric productivity [39]. A recent study compared the stress tolerance profiles of eight probiotic strains following microencapsulation challenged with acid, bile and heat stress [40]. Microencapsulated strains survived three logs CFU/ml better than free probiotic control strains. Increased survival was also observed for acid and mild heat treatment in the microencapsulated strains. Other strategies such as immobilized-cell technology [41] have also been shown to enhance the stress tolerance profiles of certain probiotic strains. However further research is required before industry standards are reached [39] and research on technologies such as microencapsulation and its benefits on the stability and release of bioactive compounds in the gastrointestinal tract [42].

### A need for alternative strategies

Whilst it has been clearly demonstrated that probiotics can be effective therapeutics in certain cases [4,8,15,21,26] results are often conflicting and can vary between and within individuals. This is due in part to different modes of action of probiotics as well as strain specific effects. Coupled with this, the increase in antibiotic resistance due to the indiscriminate use, overuse and misuse of antibiotics and the emergence of the so-called 'superbugs' (multi-antibiotic resistant strains), the need for novel and alternative therapies is paramount. The economic burden to the medical care sector in the United States for the treatment of patients with infections caused by antibiotic resistant organisms is estimated to be four billion $US annually [43]. In addition, the fact that no new antibiotic classes have been discovered [44] and that pharmaceutical companies have severely reduced investment and in some cases, completely abandoned antimicrobial research and development [45], reinforces the point that radically new and innovative therapies are urgently needed. The design, creation and genetic modification of probiotic stains exclusively tailored to target a specific pathogen or toxin thereof, or as vaccine and drug delivery vehicles is a promising and rapidly expanding area of research.

In support of this, lactobacilli possessing desirable properties such as inherent immunogenicity, bile resistance and the ability survive and proliferate in the gastrointestinal tract [46], have been successfully employed as oral, mucosal vaccine delivery vehicles. Such vaccine delivery vehicles offer an advantage over traditional live attenuated pathogenic strains in that there is no possibility of reversion to a virulent phenotype which always remains with the attenuated pathogenic strains [46]. The majority of pathogens initiate their initial infection at a mucosal surface and vaccination against such pathogens is best accomplished through mucosal vaccination. Mucosal vaccines offer a number of functional advantages [47] as well as practical benefits; they are non-invasive, easy to administer and do not require the presence of medically trained personnel, a significant advantage in the developing world [48]. Lactobacilli expressing Tetanus toxin fragment C (TTFC) have been shown to be able to elicit a positive immune response [49]. Such a strategy could be applied to the expression of other toxins from enteric pathogens, thus providing immune protection. In addition, a recombinant *Lactococcus lactis *strain producing human IL-10 has been investigated in a human clinical trial for treatment of Crohn's disease [50].

### Designer probiotics

**Figure 1:**
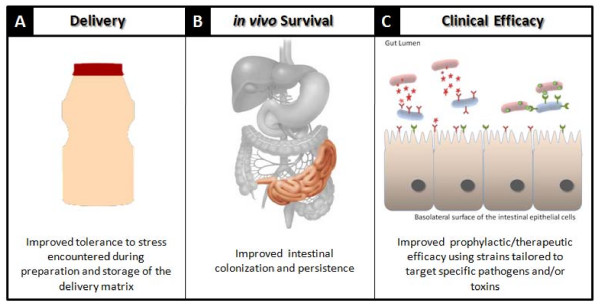
**Recent advances in the design of more effective probiotic cultures by (A) improving probiotic tolerance to stresses encountered during food manufacture and storage**. (B) Improving *in vivo *resistance to host specific stresses, thus facilitating improved gut colonization and persistence and (C) designer probiotics which specifically target pathogens and/or toxins; thus improving prophylactic and therapeutic effect [52].

As previously mentioned, diarrheal diseases are responsible for significant morbidity and mortality worldwide and treatment is becoming increasingly more difficult due to the rise in antibiotic resistance. Paton and co-workers described the development of designer probiotics for the prevention of gastrointestinal infections using a strategy involving the expression of host cell receptor-mimics on the surface of probiotic strains which can bind to the pathogen itself or neutralize and mop up secreted toxin [51,52] (Figure 1). This approach has a number of advantages; (i) the probiotic can be administered orally (ii) numerous human receptors recognized by enteric pathogens or their toxins are well characterized (iii) preventing pathogen adherence would inhibit development of infection (iv) sequestration of a toxin would prevent clinical presentation of symptoms so the pathogen can be removed by the host immune system and possibly most importantly (v) this therapeutic strategy does not apply a selective pressure on the pathogen with the of development of resistance extremely unlikely. Resistance usually results from a reduction in bacterial cells numbers due to the selective pressure and this does not occur with receptor mimic therapy.

Research in the same lab has resulted in the construction of a harmless *Escherichia coli *strain engineered to express two galactosyl-transferase genes from *Neisseria gonorrhoeae*. This resulted in a modified lipopolysaccharide (LPS) that mimics the Shiga-toxin (Stx) receptor and was found to effectively bind both Stx1 and Stx2. The engineered bacteria were shown to be 100% effective in treating mice infected with normally fatal shigatoxigenic *E. coli *(STEC) [53]. In another study by the same research group, a probiotic was developed for the treatment of travellers' diarrhea caused by enterotoxigenic *E. coli *(ETEC). Using a similar strategy to that mentioned above, an *E*. *coli *strain was engineered to produce a chimeric LPS receptor mimic capable of binding a heat-labile enterotoxin. *In vitro *tests showed more than 93% of the toxin could be neutralized by the recombinant strain, while *in vivo *studies showed the recombinant strain provided protection against fluid loss due to the toxin in rabbit ligated ileal loops [54]. A molecular mimicry strategy was also used to create a recombinant probiotic for the treatment and prevention of cholera. Cholera, caused by the bacterium *Vibrio cholerae*, is a serious intestinal infection characterized by severe, watery diarrhea resulting in rapid fluid loss and dehydration which can lead to death in just a few hours without treatment. Transmission is *via *the fecal-oral route, usually due to consumption of contaminated drinking water and the disease is epidemic in many developing countries. Current treatment is administration of a standard oral rehydration solution (ORS) to replace lost fluids, salts and electrolytes in combination with antibiotics in some cases. This is usually an effective treatment but must be administered promptly following infection. A similar strategy was used to design a probiotic strain with an altered LPS which terminates in a structure that mimics the G_M1 _ganglioside terminus, which is the binding receptor for cholera toxin (CT) [55]. The bacteria were found to be able to bind CT, abolish >99% of its cytotoxicity and absorb more than 5% of its own weight of toxin. Murine studies demonstrated that administration of the recombinant probiotic immediately following infection with *V. cholerae *increased survival rates of the mice. Furthermore, when administration of the probiotic was delayed until after establishment of *V. cholerae *infection, all mice in the probiotic group survived compared to 1 out of 12 for the control group [55]. Such powerful and significant results reinforce the potential of such alternative therapies. Further research to improve and fine-tune this approach may be necessary before such therapies can be applied to humans. For example it has been suggested that the introduction of genes to aid gastric transit or promote improved gut colonization would reduce dose regimes and thus costs, while the use of food grade bacteria such as lactococci and lactobacilli for receptor mimicry may also be possible [51]. As mentioned earlier, evidence is lacking for the recommendation of probiotics for use in the treatment of *C. difficile*. However designer probiotics may offer a viable alternative [56]. The bacteriocin lacticin 3147 has been shown to have significant antibacterial activity against *C. difficile *[57] however it is acutely sensitive to gastric acidity *in vivo *[58]. This sensitivity might be overcome by cloning bacteriocin production (and resistance) genes into a suitable host, such as a lactobacillus which could survive stomach passage and deliver the intact bacteriocin to the point of infection. Furthermore, designer probiotics meet the criteria laid out by McFarland [59] for novel approaches to manage *C. difficile *[56,60-62]. With regard to inflammatory bowel disease, there have been some studies involving recombinant probiotics with some good preliminary results being seen in the treatment of induced- colitis in animal models [60-62]. However, more information is needed on the exact causes of such conditions before suitable treatments can be fully developed for use in human trials.

### Patho-biotechnology

**Figure 2:**
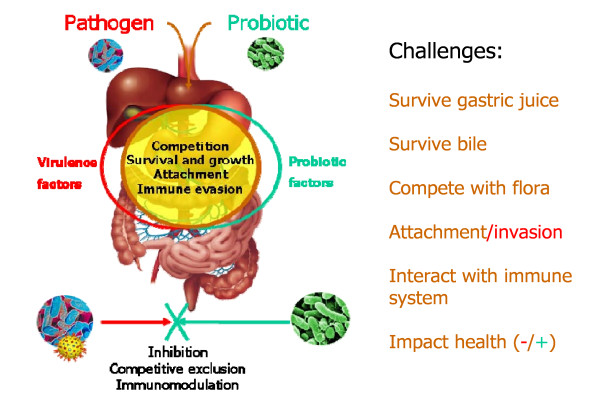
**Patho-biotechnology - the exploitation of pathogen derived virulence associated and stress survival factors for beneficial applications**. Pathogens and probiotics experience an almost identical set of challenges during gastrointestinal transit. A virulence associated factor in a pathogen may thus be exploited as a beneficial host adaptive system in a probiotic [66].

The term patho-biotechnology was coined to describe the concept of exploiting pathogenic bacteria or more precisely, exploitation of their stress adaptation, host evasion and virulence or virulence-associated characteristics for beneficial use in the biotechnology and food industries and in medicine [63]. The patho-biotechnology concept encompasses a number of different areas. Firstly, it involves the use of pathogens such as *Listeria monocytogenes *as novel vaccine and drug delivery vehicles [64]. This may be approached either by the use of conditional auxotrophic mutants or the selective elimination of key virulence factors. Secondly, such a strategy may involve the isolation of certain immunogenic proteins from specific pathogens thus removing the necessity of using the pathogen itself as the carrier vehicle [65]. The final area applicable to the patho-biotechnology approach and the main focus of this chapter is the introduction of stress survival genes from pathogenic bacteria into non-pathogenic probiotic strains [63]. Probiotic microorganisms encounter an identical set of stress conditions as pathogens upon encountering a host (Figure 2). Thus from a human point of view, an undesirable element from a pathogen (e.g. genes that aid survival in stressful conditions such as gastric acid, bile, low iron, increased osmolarity) if introduced to a probiotic could prove to be beneficial by increasing its resistance to host-associated stresses as well as its technological robustness and clinical efficacy, a distinct advantage with many potentially promising probiotic strains [34].

*L. monocytogenes *serves as an ideal candidate for the patho-biotechnology concept [66]. Its genome has been fully sequenced [67], *L. monocytogenes *is amenable to genetic manipulation [68] while physiologically it is a robust pathogen capable of resisting numerous stresses [69] while also eliciting a strong host immune response [70].

**Figure 3:**
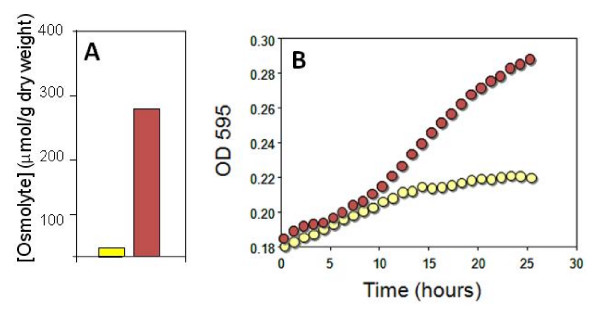
**(A) [14C]glycine betaine uptake in the *Lactobacillus salivarius *wild type (yellow bar) and the BetL complemented strain UCC118-BetL^+ ^(red bar)**. (B) Growth of *L. salivarius *wild type (yellow circles) and UCC118-BetL^+ ^(red circles) in MRS broth with 7% added NaCl [77].

The patho-biotechnology approach has been successfully employed to increase the stress tolerance of the probiotic bacterium *Lactobacillus salivarius *UCC118 [71]. *L. salivarius *has been shown to have desirable therapeutic properties [72] and has recently been shown to protect mice from *L. monocytogenes *infection by the production of the bacteriocin, Abp118 [73]. However the bacterium is technologically fragile. In an effort to improve the physiological robustness of the strain the *betL *gene of *L. monocytogenes *(encoding the compatible solute betaine uptake system, BetL) was cloned into *L. salivarius *UCC118 [71]. Bacteria accumulate compatible solutes such as betaine, either from their environment or by *de novo *synthesis, to counter osmotic stress. BetL has been shown to increase *L. monocytogenes *tolerance to salt [74], low temperature [75] and pressure stress [76], as well as increasing viability in certain foods [77]. Thus, it was postulated that the introduction of *betL *into *L. salivarius *UCC118 might improve the strain's tolerance to a number of stresses. Indeed the strain harbouring the *betL *gene (*betL*^+^) showed a significantly higher growth rate at 7% NaCl compared to the control strain lacking *bet *(*betL*^-^) (Figure 3). Also, when exposed to low temperature stress, *betL*^+ ^survival was 2 logs greater at -20°C and 0.5 logs greater at -70°C compared to wild type strain [71].

Furthermore, significantly higher survival rates for *betL*^+ ^were observed following both freeze and spray drying treatment compared to the control. Higher survival rates were also observed following high pressure processing (300 and 350 MPa). These results clearly demonstrate the potential of patho-biotechnology for improving the technological robustness of probiotic microorganisms. In addition to improving a strain's resistance to stresses encountered during food manufacture and storage, the patho-biotechnology concept has also been applied to tailor improved probiotic resistance to host specific stresses, significantly improving colonization, persistence and clinical efficacy. In support of this, the *betL *gene from *L. monocytogenes *was cloned into the probiotic bacterium *Bifidobacterium breve *UCC2003 [78]. *B. breve *UCC2003 strains expressing *betL *were shown to exhibit significantly increased tolerance to simulated gastric juice (pH 2.5) as well as osmotic stress. In addition, following successful colonization of the murine intestine, *B. breve *UCC2003 *betL*^+ ^strains were recovered at significantly higher levels in the faeces, large intestine and caecum of inoculated mice. Finally, the *B. breve *UCC2003 *betL*^+ ^strain was shown to protect against *L. monocytogenes *infection in a murine model. *L. monocytogenes *was recovered in significantly lower numbers from the spleens of mice fed with *B. breve *UCC2003 *betL*^+ ^compared the control strain (which lacked *betL*) [78]. To the best of our knowledge this study provides the first direct evidence for improved therapeutic efficacy using a patho-biotechnology based approach.

**Figure 4:**
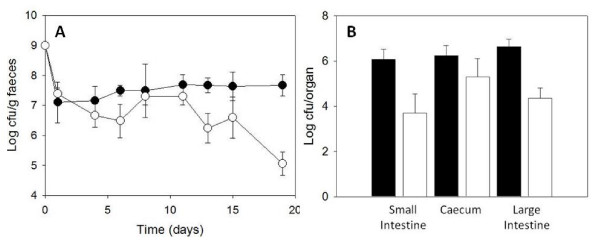
**(A) Effect of *bilE *on the gastrointestinal persistence of *Bifidobacterium breve bilE*^+ ^(black) and *Bifidobacterium breve bilE*^- ^(white) were used for peroral inoculation of female BALB/c mice (n = 5)**. *Bifidobacterium breve *counts were determined in stools at 48 hour intervals. (B) At day 19 mice were sacrificed and *Bifidobacterium breve *harbouring *bilE *(white bars) were recovered at significantly higher numbers in the intestines and caeca than the controls (black) [79].

Following these initial experiments, subsequent research was carried out in relation to improving the bile tolerance of two common probiotic strains. The authors cloned the bile exclusion system, BilE (genes *bilEA *and *bilEB*) from *L. monocytogenes *into both *L. lactis *NZ9000 and *B. breve *UCC2003 [79]. The BilE system functions as a cellular bile exclusion system and aids gastrointestinal transit and persistence in *L. monocytogenes *[80]. It was therefore postulated that heterologous expression of the BilE system would increase bile tolerance and subsequent gastrointestinal persistence of the probiotic strains. Indeed, the authors found that both *L. lactis *and *B. breve *expressing *bilE *exhibited a 2.5 logs greater survival rate compared to the wild type when grown in porcine bile at concentrations similar to that found in the intestine. Also, both *bilE*^+ ^strains persisted for longer in the gastrointestinal tract and murine faeces (Figure 4A), while *B. breve bilE*^+ ^was recovered at significantly higher numbers (2 logs greater) directly from the murine intestine compared to the wild type (Figure 4B). Furthermore *B. breve bilE*^+ ^significantly reduced *L. monocytogenes *numbers in the liver following oral infection with the pathogen. These results are significant in that increased bile tolerance also conferred increased gastrointestinal persistence which may enhance probiotic efficacy in therapeutic models [79].

An alternative approach to using pathogen-derived genetic elements to improve the physiological robustness of potential probiotics involves a relatively new and rapidly expanding area of scientific research; metagenomics.

### Metagenomics

Metagenomics is the culture-independent analysis of the collective genomes of a population of microorganisms. The field of metagenomics is relatively new, with the first international conference on the topic being held in 2003. A metagenomic analysis involves the functional and sequence-based analysis of collective microbial genomes in an environmental sample [81]. Metagenomics can be used to study as yet uncultured microbes, which represent more than 99% of the population in some environments. The total number of prokaryotic cells on earth has been estimated at 4-6 × 10^30 ^[82], with the majority of these remaining unknown to science. This diversity represents a vast genetic pool that may be utilized for the discovery of novel genes, entire metabolic pathways and their products [83]. Functional metagenomic analysis is based on the construction of clones containing metagenomic DNA in a surrogate host and subsequent screening of the clones for the expression of a desired trait or phenotype. Functional metagenomics has identified novel antibiotics [84], Na^+^/H^+ ^anitporter membrane proteins [85], esterases [86], proteases [87] and lipases [88]. Sequence based metagenomics can involve the complete sequencing of clones containing phylogenetic anchors that indicate the taxanomic group that is the probable source of the DNA fragment or it can involve the random sequencing of clones to identify a gene of interest and subsequent the search for phylogenetic anchors in the flanking DNA, which can provide a link of phylogeny with the functional gene [89]. Sequence based analysis has been used to identify a bacterial rhodopsin gene; the first evidence that rhodopsins are not exclusive to Archaea [90]. Sequenced based approaches have also been used to identify novel oxidative coupling enzymes [91], chitinases [92] and a novel fibrinolytic metalloprotease [93] to name a few.

### The human gut metagenome and meta-biotechnology

Research in our laboratory has focused on the human gut metagenome. The human distal gut is the highest density natural bacterial ecosystem known and the number of bacterial cells on or in our bodies is estimated to be 10 times greater than the number of human cells [94]. Furthermore, the number of genes in the representative species probably exceeds the number of human genes by 100-fold [95]. In this respect it has been suggested that we as humans should be considered a superorganism in symbiosis with our vast microflora [96]. This microbiome is largely untapped and contains a virtually limitless supply of novel genes to be exploited for use in medicine, science and industry. Exploiting or 'mining' the human gut metagenome for the development of novel therapeutics and designer probiotics, is essentially an extension of the patho-biotechnology concept. Considering these genes would be isolated from commensal species they are in essence 'self' genes and may alleviate some concerns regarding the use of genetic elements from pathogenic species. 'Mining' the intestinal flora to discover novel antimicrobial peptides, immunoregulatory molecules and stress tolerance genes for therapeutic purposes, is a promising and exciting area of research and a logical extension of the patho-biotechnology concept [97]. We thus coin the term *'meta-biotechnology' *to describe the use of metagenomics as a robust tool to identify novel genes for the use in biotechnology, specifically building on the patho-biotechnology concept, by increasing the technological and physiological robustness and clinical efficacy of probiotic bacteria.

As mentioned above our research has focused on the human gut metagenome and more specifically the identification of novel osmotolerance loci used by bacteria in this environment. A metagenomic library of the human gut microbiome was screened to identify clones with increased osmotolerance compared to host strain. Representative clones were subsequently subjected to transposon mutagenesis in an attempt to disrupt osmotolerance loci. We have identified a number of such genes and current work is focused on testing their potential to increase osmotolerance in other bacterial strains. The ability to cope with fluctuations in environmental osmolarity is key to the survival and viability of all microorganisms and especially so to both bacteria transiently moving through the human gastrointestinal tract and to those who permanently colonize and proliferate in that environment. Mechanisms of osmotolerance have been well characterized for the pathogenic bacterium *L. monocytogenes *(for a review see [98]) and recently, osmolyte transporters have been shown to increase bile resistance [99], an important factor in gastrointestinal persistence. We hope that the identification of novel osmotolerance loci from our host microflora using a functional metagenomic approach will enable us to create probiotic strains with an increased physiological and technological robustness, as has been demonstrated previously with genes from the pathogen *L. monocytogenes *[71,78,79].

### Biological containment

The major disadvantage with designer probiotics is that they are genetically modified organisms (GMOs) and as such their use in the treatment of humans would essentially constitute the deliberate release of such a GMO into the environment. Therefore the safety of such strains needs to be guaranteed and stringently monitored so that they do not; (i) possess antibiotic selection markers, (ii) have the ability to accumulate in the environment and (iii) transfer the genetic modification to other bacteria through lateral dissemination. Biological containment can be divided into active and passive forms. Active containment is conditionally controlled by the production of a compound that is toxic to the cells. Gene expression is tightly regulated and controlled by an environmentally responsive element. Passive containment meanwhile, is dependent on complementation of an auxotrophy by supplementation with either an intact gene or the essential metabolite (for a recent review see; [100]. Perhaps the most elegant method developed to date, which combines the advantages of both active and passive containment, is that of the thymidylate synthase gene (*thyA*) [101]. The *thyA *gene form *L. lactis*, which is essential for growth, was replaced with the expression cassette for human interleukin-10 (*hIL-10*) by double crossover using a non-replicative plasmid (pORI19). An indigenous suicide system is induced by the activation of the SOS repair system and subsequent DNA fragmentation resulting from thymine and thymidine auxotrophy. Because thymine or thymidine is essential for growth, the *thyA*-deficient strain cannot accumulate in the environment in the absence of the essential growth factors. This approach deals completely with the biosafety issues mentioned above, in that no resistance marker is required to guarantee stable inheritance of the transgene, thus removing any concerns regarding the spread of antibiotic resistance. Also, the risk of the GMO accumulating in the environment is negligible due to rapid death in the absence of thymine or thymidine. Finally, in the unlikely event that a functional *thyA *gene is acquired by homologous recombination from a closely related bacterium, the transgene would be lost [101]. One of the strains developed using this method was approved in The Netherlands for the use in a human clinical trial for the treatment of IBD. This was the first clinical trial in which live genetically modified bacteria were used as bio-therapeutics in humans [50].

## Conclusion and future outlook

Although described for over a century probiotics and research into their health benefits has only come to prominence in the past two decades. The health promoting benefits and efficacy of probiotics has been demonstrated in many models of gastrointestinal disease and indeed in diseases and conditions at other anatomically distinct locations. The use of probiotics in the treatment of many forms of diarrheal disease appears especially promising. However, in some cases results have been conflicting and large randomized, double blind, placebo controlled human trials are disappointingly rare. The inherent physiological and technological fragility of what are often promising candidate probiotic strains can render them ineffective for clinical use. Coupled with this, the alarming rise in antibiotic resistance and the emergence of many multi-drug resistant strains emphasize the need for novel thinking and approaches for the development of alternative therapeutics for the treatment of gastrointestinal disorders. Such an alternative strategy, as outlined above, involves the creation of so called designer probiotics, exclusively tailored to target a specific condition, pathogen or toxin. We have also discussed the patho-biotechnology concept and the promising results seen in a number of proof of concept studies and introduced the idea of using metagenomics to identify novel genes for use in improving the robustness of probiotic strains for the treatment of gastrointestinal disorders ('meta-biotechnology'). The development of designer probiotics will also see a reduction in production, delivery and storage costs by circumventing the short half-life and fragility associated with conventional therapeutics, a distinct advantage in the developing world. However, consumer acceptance of genetically engineered designer probiotics remains a very significant hurdle. However, it is hoped that in addition to the utilization of rigorous biological containment protocols and the application of comprehensive risk-benefit analyses, the provision of balanced objective information and consumer education on the subject as well as clearly demonstrable medical benefits will ultimately allow such therapeutics to gain a broader acceptance in the general population. With advancements in technologies and further refinements and developments in new techniques, research in this area will continue to provide novel bio-therapeutics and therapeutic targets as well as novel probiotic strains for the treatment and prevention of gastrointestinal disorders.

## Competing interests

The authors declare that they have no competing interests.

## Authors' contributions

RDS conceived of the topic. EPC, RDS and CH drafted the manuscript. All authors read and approved the final manuscript.
